# Maternal exposure to nano-titanium dioxide impedes fetal development via endothelial-to-mesenchymal transition in the placental labyrinth in mice

**DOI:** 10.1186/s12989-023-00549-3

**Published:** 2023-12-11

**Authors:** Xianjie Li, Yinger Luo, Di Ji, Zhuyi Zhang, Shili Luo, Ya Ma, Wulan Cao, Chunwei Cao, Phei Er Saw, Hui Chen, Yanhong Wei

**Affiliations:** 1 Guangdong Provincial Key Laboratory of Food, Nutrition and Health, Department of Toxicology, School of Public Health, Sun Yat-Sen University, Guangzhou, 510080 China; 2 Department of Obstetrics and Gynaecology, Sun Yat-Sen Memorial Hospital, Sun Yat-Sen University, Guangzhou, 510120 China; 3 Zhongshan People’s Hospital, Zhongshan, 528400 China; 4 Guangdong Provincial Key Laboratory of Malignant Tumor Epigenetics and Gene Regulation, Guangdong-Hong Kong Joint Laboratory for RNA Medicine, Medical Research Center, Sun Yat-Sen Memorial Hospital, Sun Yat-Sen University, Guangzhou, 510120 China; 5 Guangdong Provincial Key Laboratory of Malignant Tumor Epigenetics and Gene Regulation, Sun Yat-Sen Memorial Hospital, Sun Yat-Sen University, Guangzhou, 510120 China; 6 Department of Genetics and Cell Biology, Zhongshan School of Medicine, Sun Yat-Sen University, Guangzhou, 510080 China

**Keywords:** Titanium dioxide nanoparticle, Developmental toxicity, Endothelial-to-mesenchymal transition, Placenta, Blood vessel

## Abstract

**Background:**

Extensive production and usage of commercially available products containing TiO_2_ NPs have led to accumulation in the human body. The deposition of TiO_2_ NPs has even been detected in the human placenta, which raises concerns regarding fetal health. Previous studies regarding developmental toxicity have frequently focused on TiO_2_ NPs < 50 nm, whereas the potential adverse effects of large-sized TiO_2_ NPs received less attention. Placental vasculature is essential for maternal–fetal circulatory exchange and ensuring fetal growth. This study explores the impacts of TiO_2_ NPs (100 nm in size) on the placenta and fetal development and elucidates the underlying mechanism from the perspective of placental vasculature. Pregnant C57BL/6 mice were exposed to TiO_2_ NPs by gavage at daily dosages of 10, 50, and 250 mg/kg from gestational day 0.5–16.5.

**Results:**

TiO_2_ NPs penetrated the placenta and accumulated in the fetal mice. The fetuses in the TiO_2_ NP-exposed groups exhibited a dose-dependent decrease in body weight and length, as well as in placental weight and diameter. In vivo imaging showed an impaired placental barrier, and pathological examinations revealed a disrupted vascular network of the labyrinth upon TiO_2_ NP exposure. We also found an increase in gene expression related to the transforming growth factor-β (TGF-β) -SNAIL pathway and the upregulation of mesenchymal markers, accompanied by a reduction in endothelial markers. In addition, TiO_2_ NPs enhanced the gene expression responsible for the endothelial-to-mesenchymal transition (EndMT) in cultured human umbilical vein endothelial cells, whereas *SNAIL* knockdown attenuated the induction of EndMT phenotypes.

**Conclusion:**

Our study revealed that maternal exposure to 100 nm TiO_2_ NPs disrupts placental vascular development and fetal mice growth through aberrant activation of EndMT in the placental labyrinth. These data provide novel insight into the mechanisms of developmental toxicity posed by NPs.

**Supplementary Information:**

The online version contains supplementary material available at 10.1186/s12989-023-00549-3.

## Introduction

Titanium dioxide nanoparticles (TiO_2_ NPs) are one of the most commonly used nanomaterials [1–3]. The main routes of human exposure to TiO_2_ NP are ingestion through food supplements (E 171) and pharmaceuticals, occupational inhalation, and dermal contact with cosmetics [4–7]. A recent study has detected TiO_2_ nanoparticles in human fetal feces and found TiO_2_ (E171) in the syncytiotrophoblast microvilli and placental chorionic mesenchyme in ex vivo human placental perfusion test [8]. Moreover, experimental studies using the mouse and rat models have found that maternal exposure to TiO_2_ NPs during pregnancy can even lead to TiO_2_ NPs accumulation in the fetal brain [9, 10], suggesting that TiO_2_ NPs can penetrate the placental barrier and pose a threat to fetal health. Unfortunately, no direct epidemiological evidence exists for the link between TiO_2_ NPs exposure and adverse pregnancy or birth outcomes. Nevertheless, a longitudinal study of children in France has revealed an association between maternal occupational exposure to nanoscale particles and small for gestational age (SGA) outcomes [11]. Although there is an urgent need for epidemiological data, a large number of experimental studies on the developmental effects of TiO_2_ NPs using animals models have reported a range of impacts, including pregnancy complications, miscarriages, retarded fetal development, and aberrant breathing in the offspring [12–15]. Previous studies on the developmental toxicity of TiO_2_ have investigated fetal birth outcomes, while the early events and toxic injury mechanisms remain largely unknown.

The normal function of placental blood vessels is vital for promoting fetal growth, and placental blood vessel disturbance is often observed in adverse birth outcomes [16–19]. Previous studies on TiO_2_ NP developmental toxicity had found that exposure to TiO_2_ NPs smaller than 50 nm caused a decrease in placental diameter and weight, and a dysregulation of vascularization [20–22]. Endothelial cells (ECs) play a critical role in placental vascular growth and barrier function [23–25]. They are the central effector cells during early and long-term TiO_2_ NP exposure. However, little is known about the damaging effects of TiO_2_ NP exposure on placental vascular ECs. The endothelial-to-mesenchymal transition (EndMT) is an essential process in angiogenesis: tightly connected ECs lose their endothelial properties and acquire strong fluidity, detach from the endothelium and migrate to elongate blood vessels [26–28]. Upon hypoxia, redox stress, and inflammation, aberrant activated EndMT by the TGF-β pathway may lead to chronic vascular injury [29–31]. Given that exposure to TiO_2_ NPs can trigger oxidative stress in ECs, we hypothesized that TiO_2_ NPs may disturb fetal development through the induction of EndMT in placental blood vessels.

Previous studies have focused on developmental toxicity in TiO_2_ NPs smaller than 50 nm, while more recent attention has been paid to the effects of foodborne TiO_2_ (larger than 50 nm) on maternal and infant health [32, 33]. For instance, food-grade E171, which primarily comprises TiO_2_ particles ranging from 60 to 300 nm, with an average size of 118 nm, has recently been found to be detrimental to human health by the European Food Safety Authority (EFSA) panel [34, 35]. Animal experiments have shown that the food additive TiO_2_ (E171) can accumulate in the placenta and cause a decrease in female rats’ pregnancy rates [34, 36]. Given the increasing evidence of crossing the placental barrier, there is an urgent need to investigate whether foodborne TiO_2_ NPs (> 50 nm) impose adverse effects on fetal development and explore potential toxicity mechanisms.

In this study, we explored the adverse effects of TiO_2_ NPs (100 nm in size) on the placenta and fetal development and elucidated the underlying mechanism from the perspective of placental vasculature. We found that TiO_2_ NPs appear to interfere with vascular EndMT in the placental labyrinth, thus hindering intrauterine fetal development. Our findings may shed light on nanomaterial-induced fetal developmental toxicity.

## Results

### TiO_2_ NPs accumulate in the placenta and fetus

**Fig. 1:**
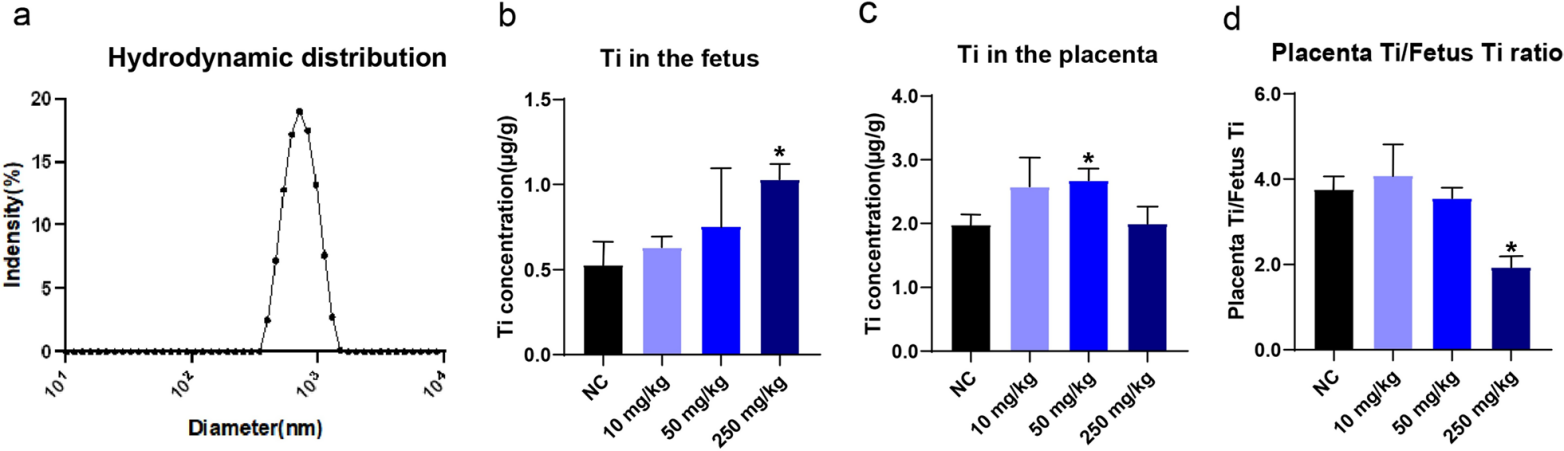
The hydrodynamic diameter of TiO_2_ and Ti concentration in the fetus and the placenta. **a** Hydrodynamic size distribution of TiO_2_; **b**, **c** Titanium element (Ti) concentrations dose-dependently elevated in the fetuses with maternal exposure to TiO_2_ NPs. Ti concentration also increased in the placenta in the 10 and 50 mg/kg groups; **d** Decreased placenta Ti/fetus Ti concentration ratios suggested Ti transportation from the placenta to the fetus, hinting disturbed placental barrier function. Data are presented as mean ± SEM. n = 3 mice or placentas. *p < 0.05 as compared with the normal control (NC)

The characterization of TiO_2_ NPs is shown in Table 1. The hydrodynamic size distribution of NPs is shown in Fig. 1a. Inductively coupled plasma-mass spectrometry (ICP-MS) has been used to measure the concentration of Ti elements to evaluate TiO_2_ NP accumulation in the placenta and the fetus. As shown in Fig. 1b, titanium element (Ti) concentrations increase in the fetus in a dose-dependent manner. Compared with a control, the Ti concentration in the fetuses increased by 19.7%, 43.1%, and 95.4% in the 10, 50, and 250 mg/kg groups, respectively (Fig. 1b). Meanwhile, Ti concentration in the placenta also increased by 30.4% and 35.3% in the 10 and 50 mg/kg groups, respectively, while no significant change was observed in the 250 mg/kg group (Fig. 1c). The ratio of placenta Ti versus fetus Ti concentration showed a decreasing tendency (Fig. 1d), hinting that TiO_2_ NPs may have penetrated the placental barrier. These data suggest that exposure to TiO_2_ NPs in size of 100 nm led to an accumulation in the placenta and a simultaneous leakage from the placenta to fetal mice.

**Table 1:** TiO_2_ NP characterization

	Primary size (nm)	Hydrodynamic diameter (nm)	Purity (%)	PDI
TiO_2_	100	630.4 ± 94.4	99	0.4

The hydrodynamic distribution of NPs were determined by dynamic light scattering (DLS) analysis using Malvern Panalytical, MA, USA. The data for each sample were obtained from three replicates.

Hydrodynamic diameter is presented as mean ± SEM. n = 3

### TiO_2_ NP exposure disrupted placental barrier function

**Fig. 2:**
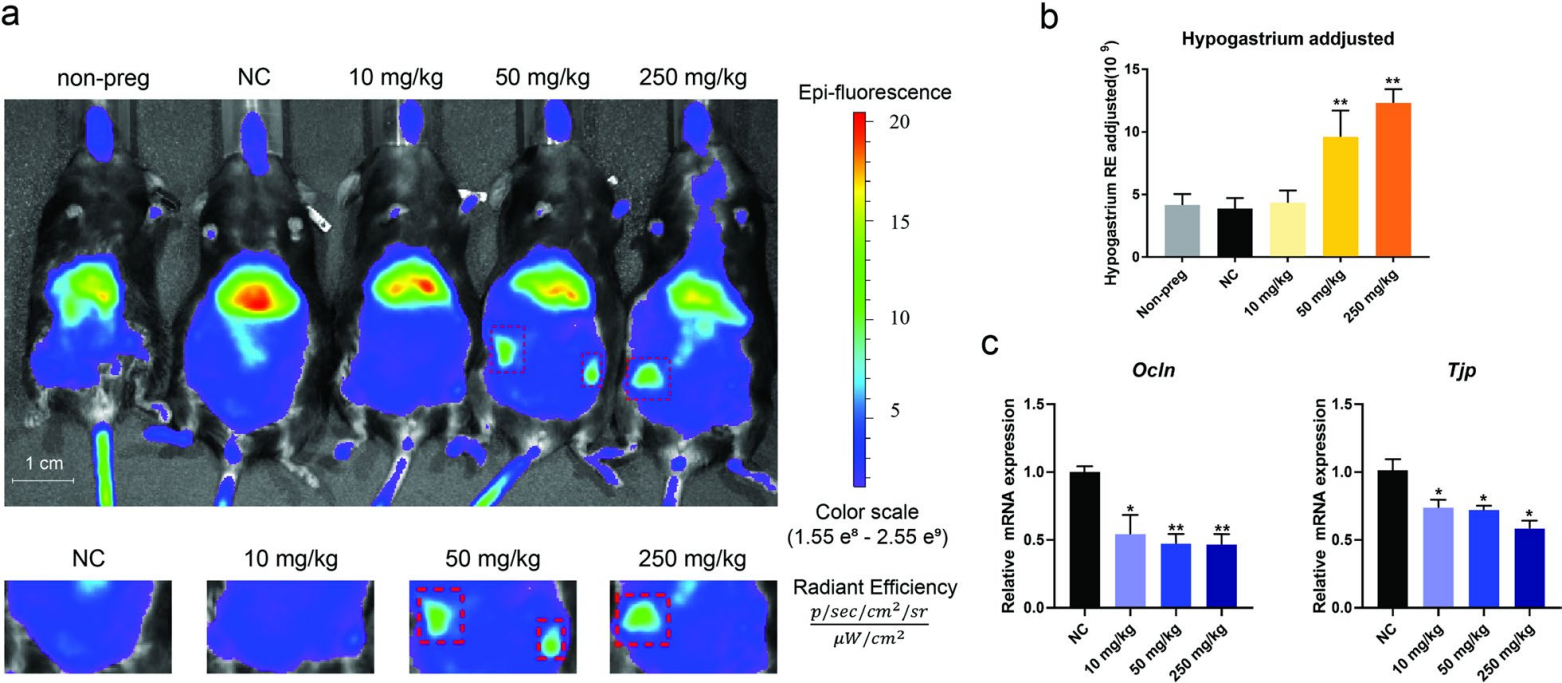
TiO_2_ NP exposure disrupted the placental barrier function. **a**, **b** We collected images with an in vivo imaging system (*IVIS*) after the indocyanine green injection. It showed stronger ICG signals in the hypogastrium in pregnant mice from the 50 and 250 mg/kg groups. Normalized by the signal intensity in the feet, the hypogastrium intensities increased in a dose-dependent manner. **c** Significant decreases of *Ocln* and *Tjp* gene expression were observed following TiO_2_ exposure, suggesting disrupted placenta barrier function. Data are presented as mean ± SEM. n = 3 mice in *IVIS*; n = 7 placentas from 7 mice in qPCR. *p < 0.05 and **p < 0.01 as compared with the normal control (NC)

A critical function of the placenta is to protect the fetus from hazards, which primarily relies on the intact placental vascular structure and function. Herein, we used the angiographic agent indocyanine green (ICG) to examine vascular permeability in mouse placentas. ICG would not penetrate through the placenta with an intact barrier and is mainly excreted by the liver. Hence it shows a strong signal in the liver. Upon barrier impairment, ICG leaks into the labyrinth and even into the fetus, represented by an increased signal in hypogastrium and a decreased signal in the liver [37, 38]. Compared with normal pregnant mice, significant increases in signal intensities in the hypogastrium were observed in the 50 and 250 mg/kg groups (Fig. 2a), whereas liver signal intensities in the TiO_2_ NP exposure group decreased slightly; after adjusting the foot signal intensities, the adjusted hypogastrium signals of the 10, 50, and 250 mg/kg exposure groups increased by 13.2%, 152.6%, and 223.7% compared with the control (Fig. 2b), respectively. This suggests that placental barrier permeability increased after TiO_2_ NP exposure. To further examine the impairment of the placental barrier, we measured barrier-related gene expression. As shown in Fig. 2c, TiO_2_ NP exposure dose-dependently suppressed gene expression of the tight junction proteins *Ocln* and *Tjp*. The result indicates that the leakage of the placental barrier caused by TiO_2_ NPs may be associated with the impaired tight junction.

### TiO_2_ NPs impeded fetal and placental growth in mice

**Fig. 3:**
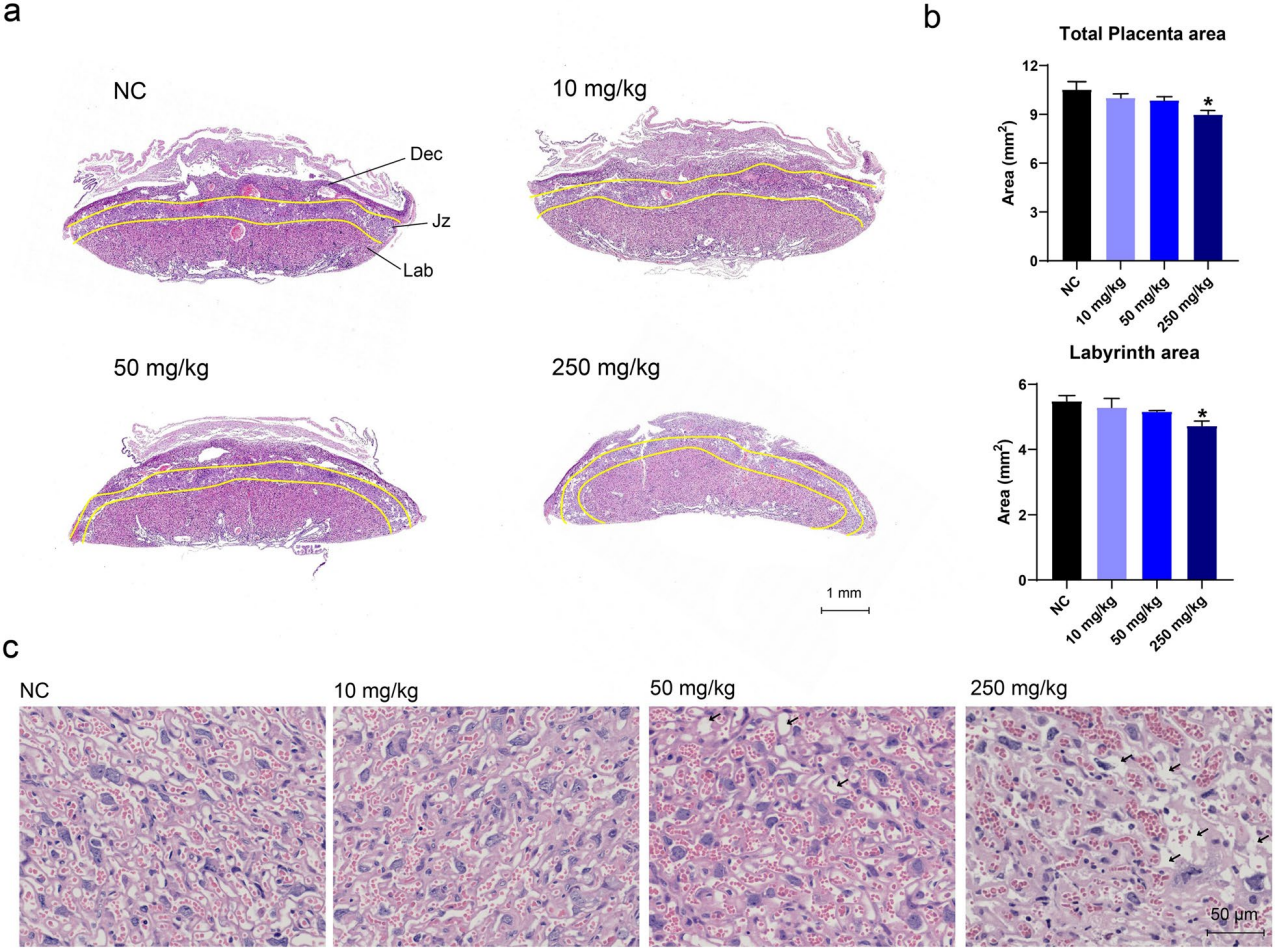
TiO_2_ NP exposure impeded placental development in mice. **a**, **b** Pathological examination revealed that TiO_2_ NP exposure decreased the placental areas and labyrinth areas. **c** TiO_2_ NP exposure led to a dose-dependent increase in vacuoles in the labyrinth. Black arrows indicate the labyrinth vacuoles. Data are presented as mean ± SEM. n = 3 placentas from 3 mice. *p < 0.05 as compared with the normal control (NC)

We observed impaired placental barrier function and TiO_2_ NP leakage to the fetuses. Hence, we evaluated the effects of TiO_2_ NPs on fetal and placental development. The numbers of fetus and fetal losses were similar between the control and exposed groups. TiO_2_ NP exposure at 10 mg/kg showed little adverse effect on the fetuses, while TiO_2_ NP exposure at 50 mg/kg and 250 mg/kg resulted in significant decreases in both fetus weights and fetus lengths (Table 2). Furthermore, placental weights decreased in a dose-dependent manner after TiO_2_ NP exposure (*p* < 0.05 or *p* < 0.01), and fetus/placenta weight ratios also decreased after TiO_2_ NP exposure. Moreover, histopathological examination showed that TiO_2_ NP exposure caused a dose-dependent decreased area of the placenta’s longitudinal section, and a pronounced reduction of the total placental area was exhibited in the 250 mg/kg group (Fig. 3a,b). Notably, the area of the placental labyrinth significantly decreased in the 250 mg/kg group. We also examined the structural change in the labyrinth (Fig. 3c) and observed a distinctly sparse structure with more and larger vacuoles after TiO_2_ NP exposure (in contrast to the compact and complex structure of the labyrinth in the control group), implicating an impeded labyrinth development. We did not observe any significant differences in organ-weight-to-body-weight ratios in maternal organs, including heart, liver, spleen, lung, and kidney (Additional file 1: Table S1). These results suggest that TiO_2_ NP exposure disrupted labyrinth structure and restrained fetal growth.

**Table 2:** TiO_2_ NP impeded fetal growth and placental development

	NC	10 mg/kg	50 mg/kg	250 mg/kg
Fetal body weight (g)	0.760 ± 0.029	0.720 ± 0.033	0.614 ± 0.041**	0.612 ± 0.030**
Crown-rump length (cm)	1.756 ± 0.019	1.717 ± 0.026	1.661 ± 0.037*	1.651 ± 0.036*
Placental weight (g)	0.129 ± 0.043	0.124 ± 0.032	0.115 ± 0.020*	0.115 ± 0.019*
Placental efficiency	6.532 ± 2.759	5.863 ± 1.671	5.219 ± 2.488**	5.538 ± 2.552*
Fetus number per dam	9.0 ± 2.2	7.3 ± 1.7	8.5 ± 1.5	8.7 ± 1.5
Total fetal loss	1	1	0	0

Data are presented as mean ± SEM. n = 51–63. *p < 0.05, **p < 0.01 as compared with the normal control (NC)

### TiO_2_ NP-induced EndMT in the placental labyrinth

**Fig. 4:**
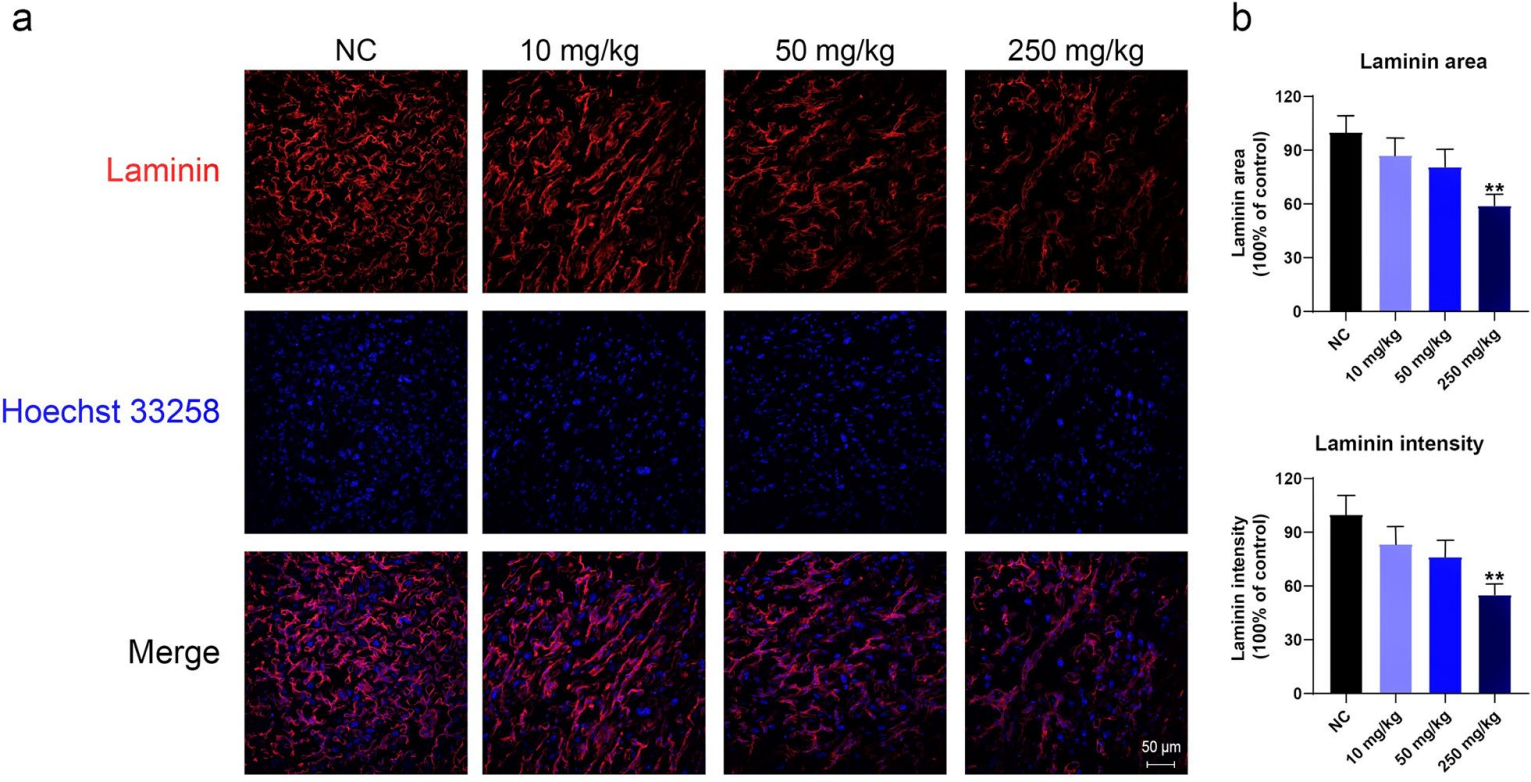
TiO_2_ NPs exposure decreased vascular density in the placenta. **a, b** Laminin was used to label the placental blood vessels. The laminin staining areas and intensities decreased after TiO_2_ NP exposure. Data are presented as mean ± SEM. n = 3 placentas from 3 mice. **p < 0.01 as compared with the normal control (NC)

As TiO_2_ NPs appear to impair placental barrier function and disturb labyrinth structure, we further examined the vasculature in the placental labyrinth. Firstly, we used laminin to label the blood vessels in the labyrinth by immunofluorescence. As shown in Fig. 4a, the laminin area in the labyrinth decreased by 14.5%, 19.5%, and 45.5% in the 10, 50, and 250 mg/kg groups, respectively. Meanwhile, the laminin intensities decreased by 20.1%, 25.6%, and 44.9% in the 10, 50, and 250 mg/kg groups, respectively. Accordingly, it appeared that the TiO_2_ NPs had affected the development of the villus blood vessels in the placental labyrinth.

**Fig. 5:**
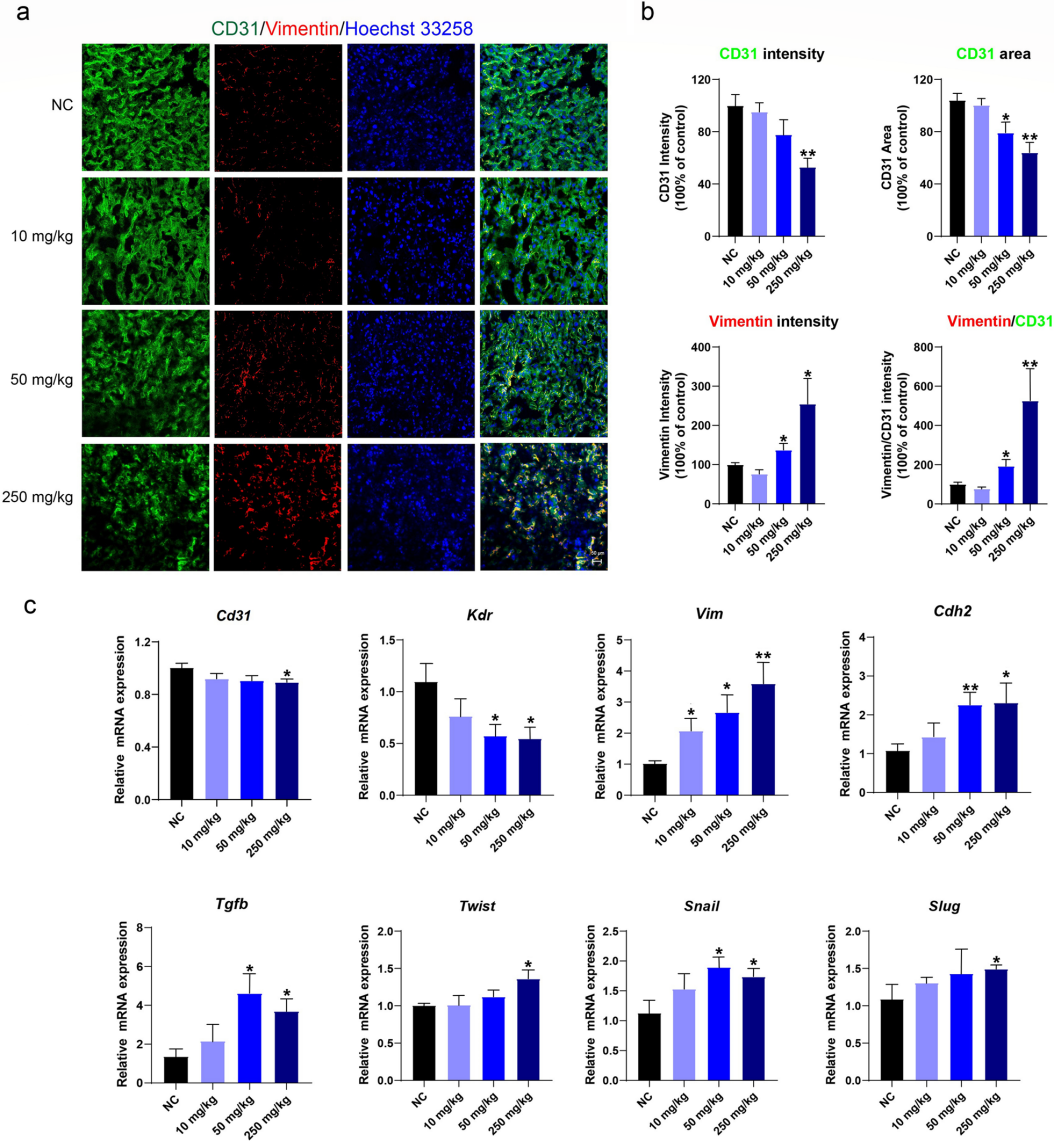
TiO_2_ NPs activated EndMT in the placental labyrinth. **a**, **b** TiO_2_ NPs exposure induced vimentin (Vim) expression and suppressed CD31 expression in the labyrinth in a dose-dependent manner. **c** The qPCR results revealed that TiO_2_ NPs activated the TGF-β-SNAIL pathway (*Tgfb, Twist, Snail, and Slug*), enhanced mesenchymal marker genes (*Vim* and *Cdh2*), and suppressed endothelial marker genes (*Kdr* and *Cd31*). Data are presented as mean ± SEM. n = 3 placentas from 3 mice for Immunofluorescence staining; n = 7 placentas from 7 mice for qPCR. *p < 0.05 and **p < 0.01 as compared with the normal control (NC)

Of note, endothelial cells (ECs) are the primary constituent cells of placental blood vessels, and endothelial dysfunction caused by aberrant activated EndMT is one of the most common causes of vascular structural disorders. Therefore, we then examined the EndMT in the placental labyrinth. As shown in Figs. 5a,b, the intensities of CD31 significantly decreased by 47.1% in the 250 mg/kg groups (*p* < 0.01); the CD31 areas also decreased by 29.5% and 42.6% in the 50 and 250 mg/kg groups, respectively (*p* < 0.05 and *p* < 0.01). We have also determined the intensity of vimentin (Vim), a typical mesenchymal marker. The result showed that the Vim intensity significantly increased by 37.0% and 154.8% in the 50 and 250 mg/kg groups, respectively. Consistently, the vimentin/CD31 intensity ratios increased by 92.4%, and 425.7% in the 50 and 250 mg/kg groups, respectively. No significant change was observed in either Vim intensity or Vim/CD31 ratio in the 10 mg/kg group. In addition, TiO_2_ NP exposure decreased the gene expressions of endothelial markers *Cd31* and *Kdr* (Fig. 5c), whereas the expressions of mesenchymal markers *Vim* and *Cdh2* increased dose-dependently (Fig. 5c). Moreover, expressions of *Tgfb*, *Twist*, *Snail,* and *Slug* increased, indicating that the transformation growth factor-β (TGF-β)-EndMT pathway had been activated and the ECs had acquired mesenchymal properties following TiO_2_ NP exposure. The results suggest that maternal exposure to TiO_2_ NPs during pregnancy induced aberrant EndMT in the placental vasculature.

### ***SNAIL*** knockdown attenuated TiO_2_ NP-induced EndMT in HUVECs

**Fig. 6:**
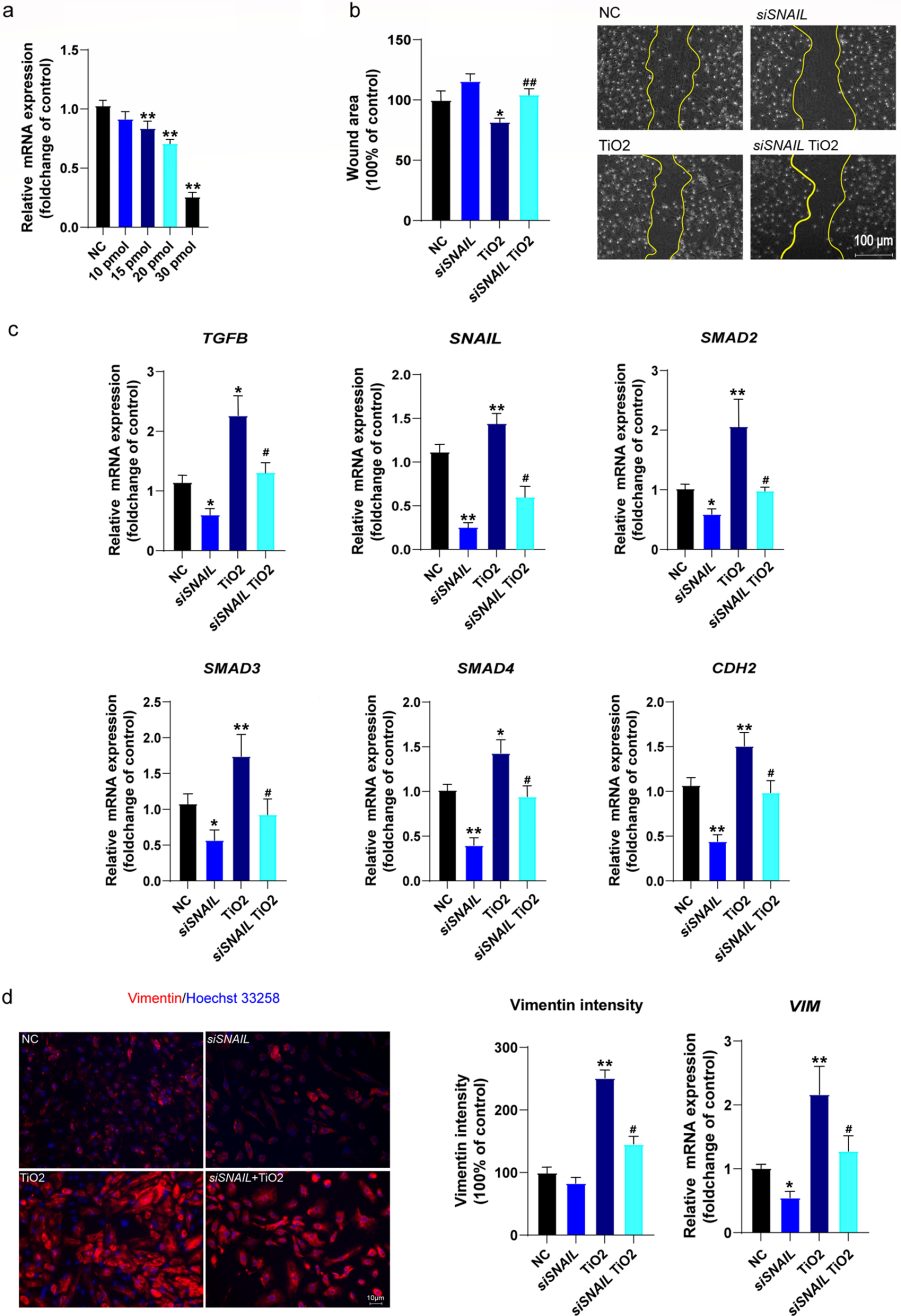
*SNAIL* knockdown attenuated TiO_2_ NP-induced EndMT in HUVECs in vitro. **a** *siSNAIL* treatment suppressed the expression of *SNAIL* in a dose-dependent manner. **b** TiO_2_ NP treatment enhanced cell migration, whereas *siSNAIL* treatment attenuated the effect. **c**, **d** The qPCR results revealed that TiO_2_ activated TGF-β-SNAIL-mediated EndMT in the HUVECs (enhanced *TGFB, SNAIL, SMAD2, SMAD3, SAMD4, CDH2,* and *VIM*), which was abrogated by knockdown of *SNAIL.* Immunofluorescence staining suggested that TiO_2_ treatment induced the mesenchymal marker vimentin in HUVECs, which was restrained by *siSNAIL* treatment. Data are presented as mean ± SEM. n = 3 independent experiments. *p < 0.05, **p < 0.01 as compared with the normal control (NC); ^#^*p* < 0.05, ^##^*p* < 0.01 as compared with the TiO_2_ group

To verify the assumption that TiO_2_ NPs disturbed the labyrinth vasculature by activating TGF-β-SNAIL-mediated EndMT, we used siRNA targeting *SNAIL* to pretreat Human Umbilical Vein Endothelial Cells (HUVECs) prior to TiO_2_ NP exposure. A scratch test was performed, EndMT gene expression was determined, and vimentin fluorescence staining was conducted to assess the mesenchymal transition of the HUVECs. As shown in Fig. 6a, *siSNAIL* treatments suppressed *SNAIL* expression of the HUVECs in a dose-dependent manner. *siSNAIL* at a concentration of 30 pmol suppressed *SNAIL* expression by 72.3%. Hence we used 30 pmol *siSNAIL* to treat the cells in the following experiment. As shown in Fig. 6b, a significantly decreased wound area by 18.3% was observed in TiO_2_ NPs treated cells compared to the control, whereas co-treatment with *siSNAIL* and TiO_2_ significantly increased the wound area by 22.7% compared to the TiO_2_ group. The gene expressions involved in the TGF-β-SNAIL-EndMT in the HUVECs were then determined (Fig. 6c, d). The mesenchymal marker genes *VIM* and *CDH2, TGFB*, *SMAD2, SMAD3,* and *SAMD4* significantly increased after TiO_2_ treatment, whereas *siSNAIL* abrogated the induction of those genes related to TGF-β-SNAIL-EndMT pathway. We also determined vimentin intensity by Immunofluorescence staining and mRNA level by qPCR. As shown in Fig. 6d, TiO_2_ NP treatment significantly increased vimentin intensity and mRNA in HUVECs compared to the control, while knockdown of *SNAIL* abolished the elevation of the vimentin level. Collectively, our results implicated that TiO_2_ NPs exposure triggered EndMT through the TGF-β-SNAIL pathway.

## Discussion

**Fig. 7:**
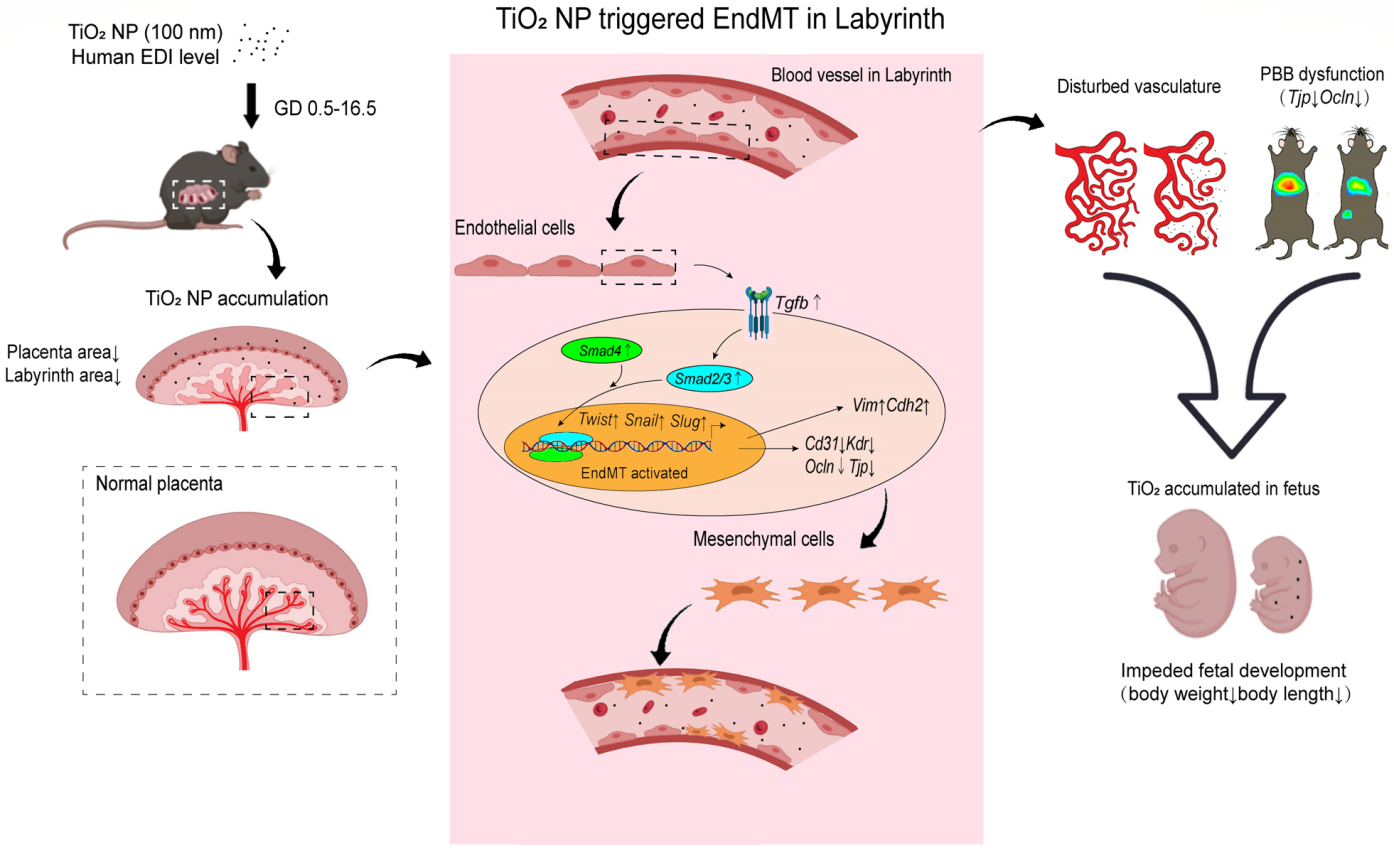
Schematic diagram of the impacts of TiO_2_ NPs on embryonic and fetal intrauterine development and the underlying mechanism. Maternal exposure to 100 nm TiO_2_ NPs resulted in placental accumulation and concomitant transfer to mouse fetuses. TiO_2_ NP exposure caused EndMT activation in the labyrinth, endothelial dysfunction, disturbance in the placental barrier, and ultimate fetal growth retardance

Usage and human exposure levels of TiO_2_ NPs have been increasing over the years, while the fetal health risks of large-sized TiO_2_ NPs above 50 nm in diameter, which are predominant portions in food additives have yet to receive sufficient attention. In the present study, we explored the adverse effects of 100 nm TiO_2_ NPs on fetal development and revealed a toxic mechanism from the perspective of placental vascular injury. We discovered that 100 nm TiO_2_ NPs triggered EndMT by activating the TGF-β-SNAIL pathway, disrupted the labyrinth vasculature in the placenta, and ultimately hindered fetal growth (Fig. 7).

We treated the pregnant mice daily with TiO_2_ of 100 nm in size by gavage from gestational day 0.5–16.5 at various concentrations, including a dose comparable to the human estimated daily intake of food-additive E171 (maximum level = 13.6 mg/kg/day). Transported in the circulation and distributed among the tissues, the majority of TiO_2_ NPs are unchanged. Nevertheless, some TiO_2_ NPs may form combination and precipitate, and very small amounts of TiO_2_ NPs would dissolve in body fluids and form ions [39]. The placental Ti concentrations in mice from the control group were comparable to basal Ti detected in human placentas[8]. Our study showed that maternal TiO_2_ NP exposure resulted in a significant accumulation of Ti in the placenta (1.32–4.37 μg/g) and the fetus (2.18–3.08 μg/g), consistent with the Ti accumulation in the fetus and the placenta reported in previous experimental studies upon exposure to TiO_2_ ranging from 10 to 35 nm (up to 5.092 μg/g in the placenta, up to 3.259 μg/g in the fetus). We found that the fetal Ti concentration increased in the 10, 50, and 250 mg/kg groups in a dose–response manner. In addition, Ti concentration dose-dependently increased in the placenta in the 10 and 50 mg/kg groups, while it did not change in the 250 mg/kg group. Notably, the highest placental barrier leakage in the ICG assay, the most disorganized vasculature, and the most robust EndMT activation were observed in the 250 mg/kg group. These results suggest that the vasculature is severely disrupted in the placenta exposed to 250 mg/kg TiO_2_ NPs, which may lead to an accentuated TiO_2_ leakage from the placenta to the fetus and ultimately alleviates placental Ti accumulation.

The placental barrier plays a critical role in maintaining fetal health [40]. In this study, we assessed placenta barrier function by in vivo imaging system (*IVIS*) using clinical angiographic agent ICG. Our findings showed that the placental barrier was significantly impaired in mice exposed to 50 and 250 mg/kg TiO_2_ NPs at GD 13.5. This may have been the reason for TiO_2_ NP leakage to the fetuses. In addition, the expression of barrier function genes *Ocln and Tjp* in the placenta also decreased, consistent with previous reports of blood barrier disruption due to nanomaterials [41–43]. Previous studies usually assessed the effect of nanoparticles on placental barrier function using the histopathological examination or detection of tight junction protein expression [44, 45]. Actually, *IVIS* offers a more readily approach for the in vivo placenta function evaluation in NP-induced developmental toxicity. The blood-placental barrier consists of endothelial cells of villi blood vessels, trophoblasts, and a thin layer of connective tissue [46]. In our study, TiO_2_ NP exposure caused the downregulated gene expression correlated with the vascular barrier, disordered vascular network, and aberrant activated EndMT in the placenta. The results strongly suggest that placental vasculature is a critical target of TiO_2_ NPs_,_ and vascular disruption is more likely to be an essential reason for the placental barrier impairment. This raises further concerns about nanoparticles larger than 50 nm in the placental vascular system.

We then examined TiO_2_ NPs’ effects on fetal development following impaired placental barrier function. The impaired placental barrier perturbed fetal development in the 50 and 250 mg/kg groups. Similar to TiO_2_ NPs smaller than 50 nm [47], the developmental toxicity of 100 nm TiO_2_ NPs to fetal mice was manifested primarily as fetal growth. Interestingly, a previous study treated rats with 153 nm TiO_2_ NPs at 1000 mg/kg from GD 6 to GD 20 did not induce maternal and embryo-fetal developmental toxicities [48]. The distinct response may be due to TiO_2_ size difference and/or a strain-dependent effect. Alternatively, the exposure window may play a decisive role since the placental development begins at GD 3.5 [49, 50], and GD 3.5 and GD 6 is probably more sensitive to TiO_2_ exposure. Moreover, our study is the first to show that maternal exposure to 100 nm TiO_2_ NP during pregnancy impedes placental growth. The decreased placental size and disordered vascular network indicate a compromised placental development and a decrease of terminal villi blood vessels (increased vacuoles), which lower the maternal–fetal exchange efficiency, cause insufficient nutrient and oxygen transfer [51, 52], and eventually impede fetal growth caused by TiO_2_ NPs exposure. The dose-dependent decrease in laminin staining areas and intensities also suggested the severely impeded vascular development in the labyrinth. Defects and deficiencies in labyrinth formation are frequent causes of developmental failure and growth deficits [53, 54]. Previous studies found that TiO_2_ smaller than 25 nm induced placental labyrinth vascular structural disorder at the level of 10 mg/kg. Given that labyrinth development in mice begins at GD 8.5 [55–57], we speculate that placental labyrinth vascular impairment may be an early event in TiO_2_ NPs’ developmental toxicity.

Aberrant activation of EndMT by xenobiotics and stress can cause endothelial dysfunction, which leads to disordered vascular structure [58–61]. In the present study, we observed TiO_2_ NP decreased CD31 expression whereas increased vimentin expression in the vasculature of labyrinth, and the vimentin/CD31 signal intensity ratio increased dose-dependently. Meanwhile, the increased EndMT-related genes and mesenchymal markers (*Tgfb, Twist, Snail, Slug*, *Vim,* and *Cdh2*), and the decreased endothelial markers (*Cd31* and *Kdr)* are hallmarks of TGF-β-SNAIL-mediated EndMT. Given the increased mesenchymal markers in ECs and the correlation between EndMT-associated genes and mesenchymal EC phenotype, we speculated that TiO_2_ NPs induce the mesenchymal transition of endothelial cells in the labyrinth. This may be an essential event for TiO_2_ NPs to cause abnormal placental vascular structure and function, ultimately affecting fetal development. To date, there has been little research on EndMT in placental vascular injury, and our findings may further broaden the connection between placental EndMT and fetal development. It should be noted that EndMT-associated genes *Vim*, *Cdh2*, *Tgfb*, *Slug*, *Snail*, and *Twist* may also be expressed by trophoblasts during migration. As we extracted the whole labyrinth and quantified the mRNA levels by qPCR, we can not exclude the role of trophoblast in the regulation of those genes and may further explore it in future studies.

As *SNAIL* is known as a key regulator of EndMT [28, 62], we pretreated HUVEC cells with siRNA targeting *SNAIL* to verify the essential role of SNAIL-mediated EndMT in TiO_2_ NP-induced endothelial dysfunction. After treatment with TiO_2_ NPs, the cell migration ability of EC treated with TiO_2_ NPs is enhanced, consistent with previous studies [63, 64]. In addition, mesenchymal marker genes (*VIM*, *CDH2*) and EndMT pathway genes (*TGFB, SNAIL, SMAD2, SMAD 3, and SMAD4*) had increased, suggesting that TiO_2_ NP treatment had induced mesenchymal transition of the HUVECs. In contrast, *siSNAIL* pretreatment appeared to attenuate TiO_2_-induced EndMT. A previous study also reported that 10 nm iron oxide nanoparticles induce reversible EndMT of HUVECs at acutely non-cytotoxic concentrations [65]. In line with animal and cell experiments, we speculate that excessive EndMT activation may be responsible for endothelial dysfunction induced by TiO_2_ NPs.

We defined 10 mg/kg as a 'human-relevant dose' because the European Food Safety Authority (EFSA) calculated the estimated daily intake (EDI) of TiO_2_ NPs from food in ordinary people up to 13.6 mg/kg/day [66]. Although fetal development, ICG permeability, labyrinth area, laminin intensity, Vimentin/CD31 ratio were not significantly changed, the mRNA levels of tight junction molecules *Ocln* and *TjP*, and mesenchymal marker *Vim* were altered in the 10 mg/kg group, indicating a disturbance of gene expression related to EndMT and barrier in labyrinth vasculature. Regarding the extrapolation of our data to humans, a variety of aspects including exposure duration, interspecies differences in exposure and pharmacokinetics, and expected differences among members of the target human population (e.g. differences between adults and children) should be considered [67]. It is noted that women may expose to TiO_2_ NPs before pregnancy and human pregnancy duration (~ 40 weeks) is over ten times of the mice (~ 3 weeks). A more prolonged exposure inevitably increases the risk of developmental toxicity. Given that an explicit model or an uncertainty factor (UF) should be applied in the extrapolation, the apparent dose-dependent responses of placental impairment and fetal development with an emphasis on vasculature upon exposure to 10 ~ 250 mg/kg TiO_2_ NPs in our findings strongly indicate a health risk for human exposure. Moreover, despite the lack of EDI of the occupational population who has a relatively higher chance of being exposed to TiO_2_ NPs materials/products [68, 69], the presumable exposure range for specific TiO_2_ manufacturing workers (e.g. surface treaters and packers) might exceed 13.6 mg/kg/day. Taken together, our study provides valuable data for health risk assessment of TiO_2_ NPs’ developmental effects. Our study may extend the knowledge of nanoparticle developmental toxicity and raise the concern of TiO_2_ NPs in food additives.

## Conclusion

Maternal exposure to 100 nm TiO_2_ NPs from GD 0.5 to 16.5 during pregnancy inhibited development in fetal mice and induced placental labyrinth vascular injury. Combining in vivo and in vitro experiments, we found that developmental toxicity is mainly attributable to the EndMT activation in the placental labyrinth by TiO_2_ NPs. This suggests that food-derived TiO_2_ NPs may threaten human fetal intrauterine development.

## Materials and methods

### Chemicals and reagents

The anatase TiO_2_ NPs (T818939) consisting of 100 nm particles at a density of 4.26 g/mL at 25 °C were obtained from Macklin reagent (China). ICG was purchased from Sigma (USA). Isoflurane was purchased from Reward Life Science Co., Ltd. (China). Primary antibody to laminin (L9393) was purchased from Sigma-Aldrich (USA). Primary antibody to CD31 (550,274) was purchased from BD Pharmingen (USA). Primary antibody to vimentin (ab92547) was purchased from Abcam (UK). HRP binding secondary antibody was purchased from Santa Cruz (USA). Anti-rat IgG (H + L, Alexa Fluor® 555 Conjugate) and anti-mouse IgG (H + L, Alexa Fluor® 488 Conjugate) were purchased from Cell Signaling Technology (USA). Hoechst 33258 was purchased from Invitrogen (USA). Bovine serum albumin (BSA) and sodium chloride were obtained from Sigma (St.Louis, USA). Paraformaldehyde (PFA) was chased from Santa Cruz Biotechnology (California, USA).

### Animal administration

Female and male C57BL/6 mice (8–10 weeks old) were purchased from Sun Yat-sen University Laboratory Animal Center (SPF grade, Certificate No. SCXK 2020–0107). The mice were housed in a specific pathogen-free (SPF) facility under constant temperature (22–25 °C) and humidity (40–60%) conditions with a 12 h light/dark cycle, and food and water provided ad libitum.

After adaptive feeding, female mice were mated with male mice (2:1) overnight to generate pregnant mouse models. Gestational day 0.5 (GD 0.5) was determined upon the appearance of the vaginal hydrant, and the pregnant dams were immediately housed in individual cages. Pregnant C57BL/6 females were randomly assigned to one normal control group and three experimental groups (n = 10 in each group). We followed the estimated daily intake of food-additive TiO_2_ (average size 104 ± 40 nm) in human adults (1.9–13.6 mg/kg/day) from an EFSA report [34]. The mice in the experimental groups were treated daily with a TiO_2_ NP water suspension (10, 50, 250 mg/kg) by gavage from GD 0.5 to GD 16.5. The mice in the pregnancy control group were administered distilled water. At GD 16.5, the mice were sacrificed and the placentas and fetuses were harvested. Fetal weight, placental weight, fetal crown-rump length, and live fetuses per mother were recorded. All collected samples were weighed and maintained in frozen conditions (approximately − 80 °C) until further analysis. All animal studies were performed in strict accordance with the recommendations of the Ethical Committee for Animal Experimentation at the School of Public Health, Sun Yat-sen University, and following the NIH guidelines “Guide for the Care and Use of Laboratory Animals”.

### TiO_2_ size characterization and measurement

TiO_2_ nano-powder was diluted in 2% heat-inactivated serum in MilliQ water. Next, the solution was sonicated with an ultrasonic cleaner (SB-5200DT, Ningbo Scientz Biotechnology Co., Ltd, Ningbo, China) at 20% of maximum amplitude for 20 min on ice. The suspended TiO_2_ NP solution was freshly prepared before use, and vehicle control was achieved by sonication of 2% heat-inactivated serum in MilliQ water. The particles in the solution were then observed by transmission electron microscopy (Hitachi-7500; Hitachi, Ltd, Tokyo, Japan). Field emission scanning electron microscopy (Hitachi SU8010) with energy dispersive spectroscopy (Oxford X-MAN 50) (FE-SEM/EDS) was used to analyze the chemical elemental composition. The hydrodynamic diameter of the TiO_2_ NPs was determined by dynamic light scattering (DLS) using Malvern Zetasizer Nano ZS90 (Malvern Panalytical, UK). The data were calculated by three replicates for each sample.

The Titanium (Ti) element concentration was measured with ICP-MS. Placenta and fetal samples were weighed and added to a 12 mL teflon microwave digestion vessel along with 5 mL of 70% nitric acid and 1 mL of 30% hydrogen peroxide using a microwave-accelerated reaction system (MARS) express instrument (1600 W, ramp up to 150 °C over 15 min, ramp up to 180 °C over 15 min, hold at 180 °C for 20 min). When the digestion solution was concentrated to a total volume of 3 mL, 0.1 mL of cesium solution (1.0 μg/mL) was added as an internal standard. After dilution with nitric acid to 10 mL, ICP–MS (Agilent 7800, USA) was used to measure titanium concentrations in the collected samples. Instrumental operating conditions were as follows: 1550 W of radiofrequency (RF) power, a 1.0 L/min nebulizer gas flow rate, and a 0.9 L/min auxiliary gas flow rate. A standard Ti curve (0, 20, 40, 60, 80, and 300 μg/L) was determined and used for quantification, and the coefficients of determination (R^2^) for titanium exceeded 0.999. Blank samples, which consisted of solutions without the presence of tissue, were used to assess contamination during the experiments.

### In vivo imaging studies

The in vivo imaging studies were conducted in accordance with previous studies [37, 38]. In an experiment, five mice at GD13.5 each from the non-pregnant control, pregnant control, 10 mg/kg, 50 mg/kg, and 250 mg/kg groups were shaved and anesthetized via isoflurane (1–2%, v/v) on a platform kept at 37 °C. Indocyanine green (ICG) solution (injected concentration 1 mM) was prepared by adding 0.167 mg ICG to a solution consisting of 10 parts double distilled water and 2 parts filtered 2 mM phosphate buffer containing 9.3% sucrose). ICG was injected into the tail vein in a total volume of 200 μL. In vivo scans of the mice were performed with the following acquisition parameters over a time period of 30 min, (excitation filter: 745 nm, emission filter: 820 nm, exposure time: 2 s, binning factor: medium, f/Stop: 2, field of view: D).

For analysis of the in vivo ICG kinetics, regions of interest (ROIs) were drawn over the maternal livers (0.25 cm^2^) and feet (0.09 cm^2^) using Living Image 4.3.1 (PerkinElmer, Waltham, MA, USA). The feet were selected as the reference background region because they are far from the injection site, the hypogastrium and the liver (preliminary studies have shown that the latter exhibits high ICG emission), and can be clearly identified. Upon quantifying fluorescence with Living Image, the mice’s radiant efficiency (represented as (photons/sec/cm^2^/steradian)/(μW/cm^2^)) was analyzed. Average radiant efficiency units over the first 20 min are presented in this analysis.

### Pathological examination

Three mouse placentas from 3 different mice per treatment condition from separate litters of dams were sacrificed on GD 16.5. The placentas were quickly dissected on ice and fixed in 4% paraformaldehyde (PFA) solution for 24 h. Paraffin Sections (10 μm thickness) of three different placentas from each group stained with hematoxylin and eosin (H&E) were used to examine any pathological changes. Then sections were photographed using a Nikon DS-Fi1 microscope (Nikon) at 10 × objective magnification for morphological evaluation. Slides were observed and imaged with an Olympus IX73 microscope (Olympus Corporation, Japan). The areas of the whole placenta, the placental decidua, the spongiotrophoblast and the labyrinth were measured using sections in ImageJ (v. 1.48, National Institutes of Health). The average of the areas was calculated using 3 serial sections from 3 different placentas. To avoid bias, all placentas were analyzed without treatment knowledge.

### Endothelial cell culture and treatment

HUVECs (Cell Systems, USA) were cultured in endothelial growth medium-2 (EGM-2, Lonza, USA) and maintained at 37 °C with 5% CO_2_. TiO_2_ NPs of 0.4 μg/mL and 4.0 μg/mL were used to treat HUVEC. HUVECs were seeded into 96-well plates at a density of 50% confluence, and cultured for 24 h.

Scratch assays were conducted to analyze endothelial cell migration ability. HUVECs were seeded in 12-well plates, and when cells reached 100% confluence, the cell monolayer was carefully scratched with a pipette and then washed with phosphate buffered saline (PBS) three times. Images were acquired at 0 and 12 h after the cells were scratched. ImageJ software was used to evaluate the areas of the scratch.

Small interfering RNA sequences targeting human *SNAIL* (*Silencer*@Select Pre-designed siRNA, s13186, Ambion, USA, Sense:5′–3′GAAUGUCCCUGCUCCACAAtt; Antisense:5′–3′ UUGUGGAGCAGGGACAUUCgg) were transfected using Lipofectamine2000 (Invitrogen, USA), as recommended by the manufacturer’s instructions. Cells were collected for further analysis after culture.

### Immunofluorescence staining

Frozen placental sections of three placentas from three different mice in each group were fixed with 4% PFA, and incubated with blocking solution (1% bovine serum albumin (BSA) in PBS) for 1 h at room temperature. The primary antibodies anti-CD31 (1:200, BD pharmingen), anti-vimentin (1:200, abcam) and anti-laminin (1:500, Sigma-Aldrich) were incubated at 4 °C overnight. On the next day, the sections were incubated with the fluorescent dye-conjugated secondary antibodies at room temperature for 1 h. Then, the cells’ nuclei were visualized by Hoechst 33258 (Invitrogen, USA). Lastly, for the three sections from the same placenta, 18 fields were captured under a Nikon Eclipse Ti-SR fluorescent microscope (Nikon, Japan). The immunofluorescence staining area and intensity were quantified by ImageJ.

### Quantitative real-time PCR assays

Total RNA was extracted from the placental labyrinth using TRIzol reagent (Invitrogen, USA), and reverse transcribed with the PrimeScript® RT Enzyme Mix I (TaKaRa Biotech, Japan), as previously described [70–72]. We performed quantitative real-time PCR assays with an SYBR Green PCR Master Mix reagent kit (Toyobo, Japan), and added template cDNA to the reaction mixture. The qPCR profile was: denaturation at 95 °C for 30 s and 40 cycles at 3 steps: 95 °C for 30 s, 60 °C for 34 s, and 72 °C for 30 s. Gene expression was normalized to that of β-actin using the 2^−△△CT^ method, and data are presented as expressions relative to the indicated controls. Ubiquitin C (*UBC*) served as the housekeeping gene for calibrating the relative fold change, based on the CT value. The primer sequences are presented in the Additional file 2: Table S2 &S3. 

### Data analysis

Data were analyzed with SPSS 18.0. Statistical differences were analyzed by one-way Analysis of Variance (ANOVA) followed by multiple comparisons with Dunnett’s test. All results are presented as mean ± SEM, and *p* < 0.05 was considered statistically significant.

### Supplementary Information

**Additional file 1. Table S1:** Organ-weight-to-body-weight ratios of maternal mice.

**Additional file 2. Table S2 &S3:** Mouse and human gene-specific primers used in qPCR.

## Data Availability

The datasets supporting the conclusions of this article are included within the article and can be retrieved from the corresponding author upon reasonable request.

## Abbreviations

BSA: Bovine serum albumin
EDI: Estimated daily intake
EFSA: European Food Safety Authority
EGM: Endothelial growth medium
EndMT: Endothelial-to-mesenchymal transition
GD: Gestational day
H&E: Hematoxylin and eosin
HUVEC: Human umbilical vein endothelial cell
ICG: Indocyanine green
ICP-MS: Inductively coupled plasma-mass spectrometry
*IVIS*: In vivo image system
NC: Normal control
NIH: National Institutes of Health
NP: Nanoparticle
PBS: Phosphate buffered saline
PDI: Polymer dispersity index
PFA: Paraformaldehyde
ROI: Regions of interest
SPF: Specific pathogen free
Ti: Titanium element
TiO_2_: Titanium dioxide
UBC: Ubiquitin C

## References

[1] Robichaud CO, Uyar AE, Darby MR, Zucker LG, Wiesner MR (2009). Estimates of upper bounds and trends in nano-TiO_2_ production as a basis for exposure assessment. Environ Sci Technol.

[2] Shi H, Magaye R, Castranova V, Zhao J (2013). Titanium dioxide nanoparticles: a review of current toxicological data. Part Fibre Toxicol.

[3] Musial J, Krakowiak R, Mlynarczyk DT, Goslinski T, Stanisz BJ (2020). Titanium dioxide nanoparticles in food and personal care products-what do we know about their safety?. Nanomaterials.

[4] Shakeel M, Jabeen F, Shabbir S, Asghar MS, Khan MS, Chaudhry AS (2016). Toxicity of Nano-Titanium Dioxide (TiO_2_-NP) through various routes of exposure: a review. Biol Trace Elem Res.

[5] Winkler HC, Notter T, Meyer U, Naegeli H (2018). Critical review of the safety assessment of titanium dioxide additives in food. J Nanobiotechnol.

[6] Dréno B, Alexis A, Chuberre B, Marinovich M (2019). Safety of titanium dioxide nanoparticles in cosmetics. J Eur Acad Dermatol Venereol.

[7] Stapleton P (2018). Cardiovascular susceptibility in offspring after maternal engineered nanomaterial exposure. FASEB J.

[8] Guillard A, Gaultier E, Cartier C, Devoille L, Noireaux J, Chevalier L (2020). Basal Ti level in the human placenta and meconium and evidence of a materno-foetal transfer of food-grade TiO(2) nanoparticles in an ex vivo placental perfusion model. Part Fibre Toxicol.

[9] Mohammadipour A, Fazel A, Haghir H, Motejaded F, Rafatpanah H, Zabihi H (2014). Maternal exposure to titanium dioxide nanoparticles during pregnancy; impaired memory and decreased hippocampal cell proliferation in rat offspring. Environ Toxicol Pharmacol.

[10] Yamashita K, Yoshioka Y, Higashisaka K, Mimura K, Morishita Y, Nozaki M (2011). Silica and titanium dioxide nanoparticles cause pregnancy complications in mice. Nat Nanotechnol.

[11] Manangama G, Migault L, Audignon-Durand S, Gramond C, Zaros C, Bouvier G (2019). Maternal occupational exposures to nanoscale particles and small for gestational age outcome in the French Longitudinal Study of Children. Environ Int.

[12] Wu Y, Chen L, Chen F, Zou H, Wang Z (2020). A key moment for TiO(2): Prenatal exposure to TiO(2) nanoparticles may inhibit the development of offspring. Ecotoxicol Environ Saf.

[13] Hong F, Zhou Y, Zhao X, Sheng L, Wang L (2017). Maternal exposure to nanosized titanium dioxide suppresses embryonic development in mice. Int J Nanomed.

[14] Colnot E, Cardoit L, Cabirol MJ, Roudier L, Delville MH, Fayoux A (2022). Chronic maternal exposure to titanium dioxide nanoparticles alters breathing in newborn offspring. Part Fibre Toxicol.

[15] Ema M, Gamo M, Honda K (2016). Developmental toxicity of engineered nanomaterials in rodents. Toxicol Appl Pharmacol.

[16] Gaillard R, Steegers EA, Tiemeier H, Hofman A, Jaddoe VW (2013). Placental vascular dysfunction, fetal and childhood growth, and cardiovascular development: the generation R study. Circulation.

[17] Schatz F, Guzeloglu-Kayisli O, Arlier S, Kayisli UA, Lockwood CJ (2016). The role of decidual cells in uterine hemostasis, menstruation, inflammation, adverse pregnancy outcomes and abnormal uterine bleeding. Hum Reprod Update.

[18] Tran V, Weckman AM, Crowley VM, Cahill LS, Zhong K, Cabrera A (2021). The Angiopoietin-Tie2 axis contributes to placental vascular disruption and adverse birth outcomes in malaria in pregnancy. EBioMedicine.

[19] Wang X, Athayde N, Trudinger B (2004). Microvascular endothelial cell activation is present in the umbilical placental microcirculation in fetal placental vascular disease. Am J Obstet Gynecol.

[20] Liu Z, Zhang M, Han X, Xu H, Zhang B, Yu Q (2016). TiO2 nanoparticles cause cell damage independent of apoptosis and autophagy by impairing the ROS-scavenging system in Pichia pastoris. Chem Biol Interact.

[21] Zhang L, Xie X, Zhou Y, Yu D, Deng Y, Ouyang J (2018). Gestational exposure to titanium dioxide nanoparticles impairs the placentation through dysregulation of vascularization, proliferation and apoptosis in mice. Int J Nanomed.

[22] Abukabda AB, Bowdridge EC, McBride CR, Batchelor TP, Goldsmith WT, Garner KL (2019). Maternal titanium dioxide nanomaterial inhalation exposure compromises placental hemodynamics. Toxicol Appl Pharmacol.

[23] Sáez T, de Vos P, Kuipers J, Sobrevia L, Faas MM (2018). Fetoplacental endothelial exosomes modulate high d-glucose-induced endothelial dysfunction. Placenta.

[24] Claesson-Welsh L, Dejana E, McDonald DM (2021). Permeability of the endothelial barrier: identifying and reconciling controversies. Trends Mol Med.

[25] Schossleitner K, Rauscher S, Gröger M, Friedl HP, Finsterwalder R, Habertheuer A (2016). Evidence that cingulin regulates endothelial barrier function in vitro and in vivo. Arterioscler Thromb Vasc Biol.

[26] Bischoff J (2019). Endothelial-to-mesenchymal transition. Circ Res.

[27] Piera-Velazquez S, Jimenez SA (2019). Endothelial to mesenchymal transition: role in physiology and in the pathogenesis of human diseases. Physiol Rev.

[28] Sun JX, Chang TF, Li MH, Sun LJ, Yan XC, Yang ZY (2018). SNAI1, an endothelial-mesenchymal transition transcription factor, promotes the early phase of ocular neovascularization. Angiogenesis.

[29] Pan JA, Zhang H, Lin H, Gao L, Zhang HL, Zhang JF (2021). Irisin ameliorates doxorubicin-induced cardiac perivascular fibrosis through inhibiting endothelial-to-mesenchymal transition by regulating ROS accumulation and autophagy disorder in endothelial cells. Redox Biol.

[30] Li Y, Lui KO, Zhou B (2018). Reassessing endothelial-to-mesenchymal transition in cardiovascular diseases. Nat Rev Cardiol.

[31] Kovacic JC, Dimmeler S, Harvey RP, Finkel T, Aikawa E, Krenning G (2019). Endothelial to mesenchymal transition in cardiovascular disease. J Am Coll Cardiol.

[32] Sitia G, Fiordaliso F, Violatto MB, Alarcon JF, Talamini L, Corbelli A (2022). Food-grade titanium dioxide induces toxicity in the nematode caenorhabditis elegans and acute hepatic and pulmonary responses in mice. Nanomaterials.

[33] Weir A, Westerhoff P, Fabricius L, Hristovski K, von Goetz N (2012). Titanium dioxide nanoparticles in food and personal care products. Environ Sci Technol.

[34] Younes M, Aquilina G, Castle L, Engel KH, Fowler P, Frutos Fernandez MJ (2021). Safety assessment of titanium dioxide (E171) as a food additive. Efsa j.

[35] Younes M, Aggett P, Aguilar F, Crebelli R, Dusemund B, Filipič M (2018). Evaluation of four new studies on the potential toxicity of titanium dioxide used as a food additive (E 171). Efsa J.

[36] Bischoff NS, de Kok TM, Sijm D, van Breda SG, Briedé JJ, Castenmiller JJM (2020). Possible adverse effects of food additive E171 (titanium dioxide) related to particle specific human toxicity, including the immune system. Int J Mol Sci..

[37] Bishara A, Meir M, Portnoy E, Shmuel M, Eyal S (2015). Near infrared imaging of indocyanine green distribution in pregnant mice and effects of concomitant medications. Mol Pharm.

[38] Meir M, Bishara A, Mann A, Udi S, Portnoy E, Shmuel M (2016). Effects of valproic acid on the placental barrier in the pregnant mouse: optical imaging and transporter expression studies. Epilepsia.

[39] Mbanga O, Cukrowska E, Gulumian M (2022). Dissolution of titanium dioxide nanoparticles in synthetic biological and environmental media to predict their biodurability and persistence. Toxicol In Vitro.

[40] Megli CJ, Coyne CB (2022). Infections at the maternal-fetal interface: an overview of pathogenesis and defence. Nat Rev Microbiol.

[41] Wang DP, Wang ZJ, Zhao R, Lin CX, Sun QY, Yan CP (2020). Silica nanomaterials induce organ injuries by Ca(2+)-ROS-initiated disruption of the endothelial barrier and triggering intravascular coagulation. Part Fibre Toxicol.

[42] Wang LM, Wang YT, Yang WX (2021). Engineered nanomaterials induce alterations in biological barriers: focus on paracellular permeability. Nanomedicine.

[43] Sharma A, Feng L, Muresanu DF, Sahib S, Tian ZR, Lafuente JV (2021). Manganese nanoparticles induce blood-brain barrier disruption, cerebral blood flow reduction, edema formation and brain pathology associated with cognitive and motor dysfunctions. Prog Brain Res.

[44] Wang Z, Zhang C, Liu X, Huang F, Wang Z, Yan B (2020). Oral intake of ZrO(2) nanoparticles by pregnant mice results in nanoparticles' deposition in fetal brains. Ecotoxicol Environ Saf.

[45] Chu M, Wu Q, Yang H, Yuan R, Hou S, Yang Y (2010). Transfer of quantum dots from pregnant mice to pups across the placental barrier. Small.

[46] Vähäkangas K, Myllynen P (2009). Drug transporters in the human blood-placental barrier. Br J Pharmacol.

[47] Hougaard KS, Jackson P, Jensen KA, Sloth JJ, Löschner K, Larsen EH (2010). Effects of prenatal exposure to surface-coated nanosized titanium dioxide (UV-Titan). A study in mice. Part Fibre Toxicol..

[48] Warheit DB, Boatman R, Brown SC (2015). Developmental toxicity studies with 6 forms of titanium dioxide test materials (3 pigment-different grade & 3 nanoscale) demonstrate an absence of effects in orally-exposed rats. Regul Toxicol Pharmacol.

[49] Hemberger M, Hanna CW, Dean W (2020). Mechanisms of early placental development in mouse and humans. Nat Rev Genet.

[50] Malassiné A, Frendo JL, Evain-Brion D (2003). A comparison of placental development and endocrine functions between the human and mouse model. Hum Reprod Update.

[51] Jozwik M, Pietrzycki B, Jozwik M, Anthony RV (2009). Expression of enzymes regulating placental ammonia homeostasis in human fetal growth restricted pregnancies. Placenta.

[52] Salavati N, Smies M, Ganzevoort W, Charles AK, Erwich JJ, Plösch T (2018). The possible role of placental morphometry in the detection of fetal growth restriction. Front Physiol.

[53] Kalisch-Smith JI, Simmons DG, Pantaleon M, Moritz KM (2017). Sex differences in rat placental development: from pre-implantation to late gestation. Biol Sex Differ.

[54] Yamakage S, Oe Y, Sekimoto A, Obata H, Yasuta M, Sato E (2020). Protease-activated receptor 2 contributes to placental development and fetal growth in mice. Thromb Res.

[55] Simmons DG, Natale DR, Begay V, Hughes M, Leutz A, Cross JC (2008). Early patterning of the chorion leads to the trilaminar trophoblast cell structure in the placental labyrinth. Development.

[56] Cross JC, Nakano H, Natale DR, Simmons DG, Watson ED (2006). Branching morphogenesis during development of placental villi. Differentiation.

[57] Anson-Cartwright L, Dawson K, Holmyard D, Fisher SJ, Lazzarini RA, Cross JC (2000). The glial cells missing-1 protein is essential for branching morphogenesis in the chorioallantoic placenta. Nat Genet.

[58] Souilhol C, Harmsen MC, Evans PC, Krenning G (2018). Endothelial-mesenchymal transition in atherosclerosis. Cardiovasc Res.

[59] Gorelova A, Berman M, Al GI (2021). Endothelial-to-mesenchymal transition in pulmonary arterial hypertension. Antioxid Redox Signal.

[60] Pérez L, Muñoz-Durango N, Riedel CA, Echeverría C, Kalergis AM, Cabello-Verrugio C (2017). Endothelial-to-mesenchymal transition: cytokine-mediated pathways that determine endothelial fibrosis under inflammatory conditions. Cytokine Growth Factor Rev.

[61] Hulshoff MS, Xu X, Krenning G, Zeisberg EM (2018). Epigenetic regulation of endothelial-to-mesenchymal transition in chronic heart disease. Arterioscler Thromb Vasc Biol.

[62] Cabrerizo-Granados D, Peña R, Palacios L, Carrillo-Bosch L, Lloreta-Trull J, Comerma L (2021). Snail1 expression in endothelial cells controls growth, angiogenesis and differentiation of breast tumors. Theranostics.

[63] Brammer KS, Oh S, Gallagher JO, Jin S (2008). Enhanced cellular mobility guided by TiO_2_ nanotube surfaces. Nano Lett.

[64] Park J, Bauer S, Schmuki P, von der Mark K (2009). Narrow window in nanoscale dependent activation of endothelial cell growth and differentiation on TiO_2_ nanotube surfaces. Nano Lett.

[65] Wen T, Du L, Chen B, Yan D, Xu H (2019). Iron oxide nanoparticles induce reversible endothelial-to-mesenchymal transition in vascular endothelial cells at acutely non-cytotoxic concentrations. Part Fibre Toxicol.

[66] EFSA. Re‐evaluation of titanium dioxide (E171) as a food additive. 2016;14:9. 10.2903/j.efsa.2016.4545.

[67] OEHHA. Air toxics hot spots risk assessment guidelines technical support document for the derivation of noncancer reference exposure levels California Environmental Protection Agency 2008:131

[68] Liao CM, Chiang YH, Chio CP (2008). Model-based assessment for human inhalation exposure risk to airborne nano/fine titanium dioxide particles. Sci Total Environ.

[69] Boffetta P, Soutar A, Cherrie JW, Granath F, Andersen A, Anttila A (2004). Mortality among workers employed in the titanium dioxide production industry in Europe. Cancer Causes Control.

[70] Zhong X, Qiu J, Kang J, Xing X, Shi X, Wei Y (2019). Exposure to tris(1,3-dichloro-2-propyl) phosphate (TDCPP) induces vascular toxicity through Nrf2-VEGF pathway in zebrafish and human umbilical vein endothelial cells. Environ Pollut.

[71] Zhong X, Kang J, Qiu J, Yang W, Wu J, Ji D (2019). Developmental exposure to BDE-99 hinders cerebrovascular growth and disturbs vascular barrier formation in zebrafish larvae. Aquat Toxicol.

[72] Xing X, Kang J, Qiu J, Zhong X, Shi X, Zhou B (2018). Waterborne exposure to low concentrations of BDE-47 impedes early vascular development in zebrafish embryos/larvae. Aquat Toxicol.

